# Norwegian Scabies in Two Immune-Compromised Patients: A Case Report

**Published:** 2019-06

**Authors:** Mahmoud RAHDAR, Sharif MARAGHI

**Affiliations:** 1. Department of Parasitology, School of Medicine, Ahvaz Jundishapur University of Medical Sciences, Ahvaz, Iran; 2. Abadan Arvand International Division, Ahvaz Jundishapur University of Medical Sciences, Ahvaz, Iran; 3. Infectious and Tropical Diseases Research Center, Ahvaz Jundishapur University of Medical Sciences, Ahvaz, Iran; 4. Thalassemia and Hemoglobinopathy Research Center, Ahvaz Jundishapur University of Medical Sciences, Ahvaz, Iran

**Keywords:** *Sarcoptes scabiei*, Norwegian scabies, Renal transplant, Diabetes mellitus

## Abstract

Norwegian scabies (hyperkeratosis scabies) is an acute form of skin disease seen in immune-compromised patients. This study aimed to describe two cases of Norwegian scabies from Ahvaz, southwest of Iran in 2015. Two patients included a 55 year old man with renal transplant history and a 49 yr old man with diabetic mellitus and autoimmune disease, complained of dermatitis lesions and itching with sever hyperkeratosis, several macula and papules on neck and armpits for one-month duration were referred to a Iran Zamin Medical Diagnostic Laboratory in Ahvaz, Southwestern Iran in 2015. Patients were referred for fungal examination. Scraping from the crusted lesions of skin and slide preparation with 20% KOH was done. Microscopic examination presented that huge infestation of *Sarcoptes scabiei* in all forms of parasite included adult female, nymph stage and eggs. One of the patients spouse was also infested by *Sarcoptes* and appeared mild clinical symptoms. The disease was diagnosed with Norwegian scabies and the patients were successfully treated with topical 5% permethrin ointment for two weeks continuously. Overall, Norwegian scabies should be considered in immune- compromised patients with contaminated areas.

## Introduction

Human scabies is caused by an ectoparasite mite called *Sarcoptes scabiei* var. *hominis* belonged to Stigmata order. *Sarcoptes* mites’ barrow skin and lay several eggs in the canals and then nymph stages leave the canals and appear in surfaces for few days. In this time they can easily be transmitted to other persons who have close contact with infected one.

The disease has been recognized by itching dermatitis. Scabies has been reported from many parts of the world especially in developing countries. The main factors for distribution of the disease are overcrowding, poor personal hygiene, rural areas and ignoring to hygienic principles (1). Norwegian scabies (hyperkeratosis scabies) is an acute form of the disease seen in immune-compromised patients. Patients with immune system suppression, autoimmune diseases, malnutrition status, malignancy, organ transplantation, and HIV were more sensitive to acquire the infestation (2). Suppression of immune system allows *Sarcoptes* mite for rapidly distribution in body and presents a generalized infestation. Clinical signs included hyperkeratosis and skin crusting, severe itching and forming secondary infection that causes septicemia and mortality in untreated patients (3).

Here we report two cases of Norwegian scabies from Ahvaz, Southwest of Iran in 2015.

## Case Report

**Fig. 1:**
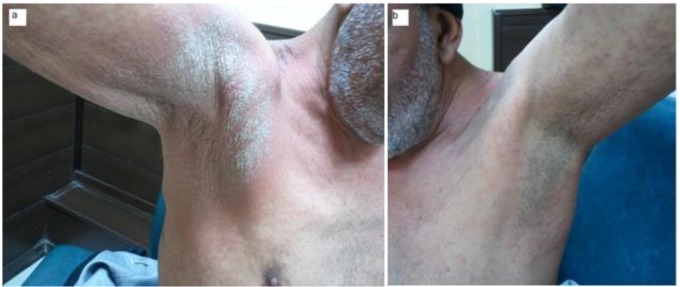
Scabies lesions in immune- compromised patient, in Ahvaz city, Southwest Iran in 2015 (a: Right armpit, b: left armpit)

**Fig. 2:**
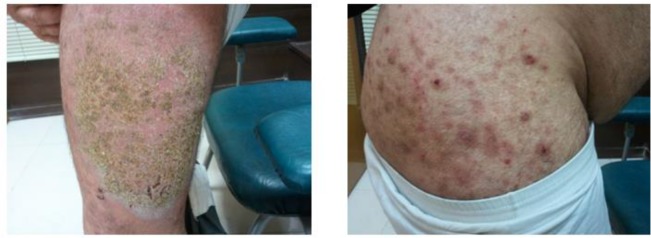
Scabies lesions in a diabetes patient, in Ahvaz city, Southwest Iran, in 2015

**Fig. 3:**
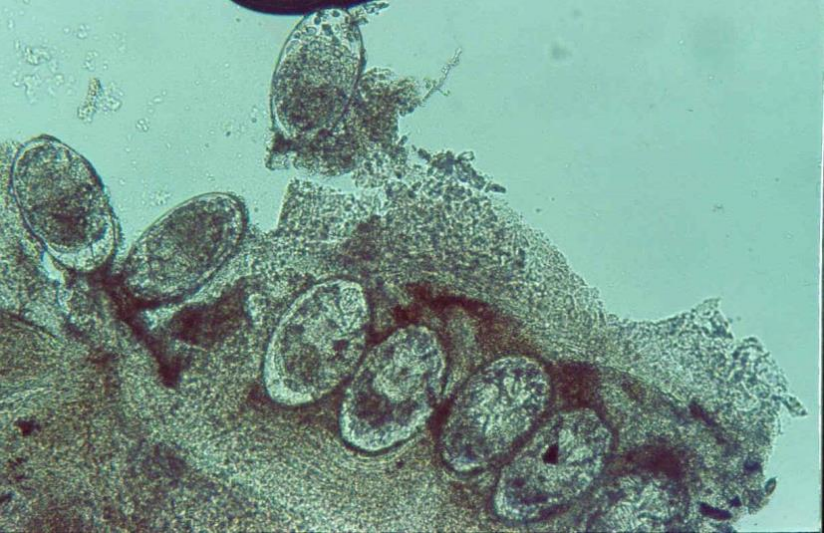
Eggs of *Sarcoptes scabiei* in the skin lesion, in Ahvaz city, Southest Iran, 2015

**Fig. 4:**
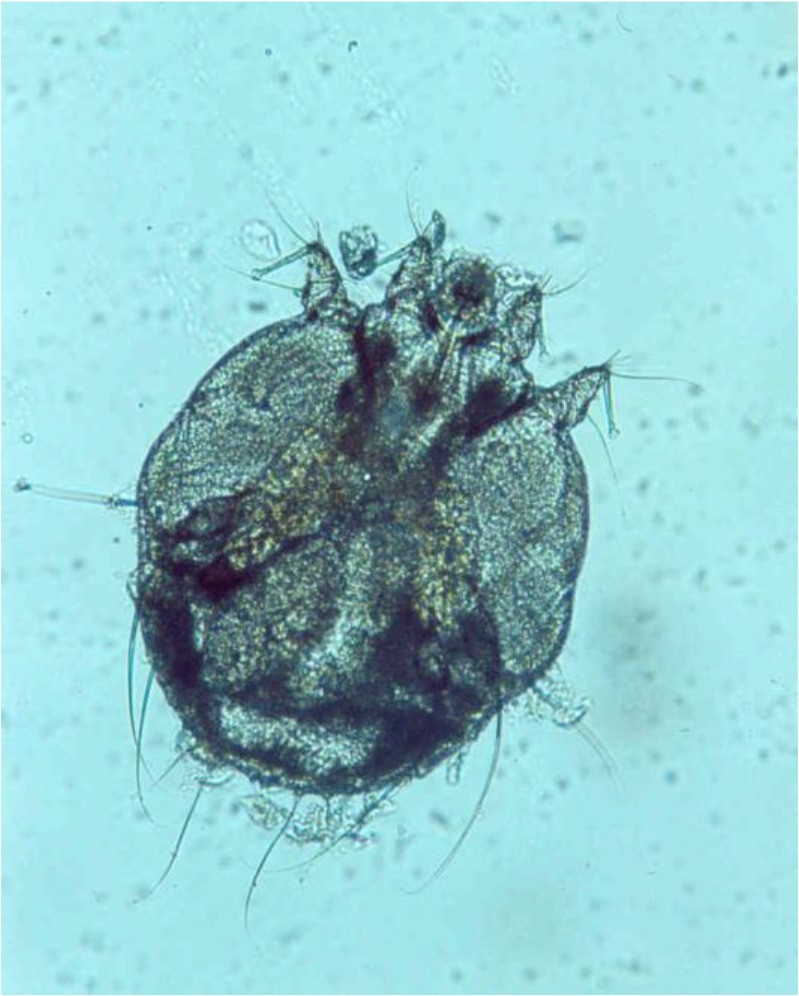
*Sarcoptes scabiei* female isolated by scraping the skin, in Ahvaz city, Southwest Iran, in 2015

Two patients were reported in this study. The first was a 55 yr old man with renal transplant history, who was taken 1000 mg prednisolone daily for one year. He complained of dermatitis lesions and itching with sever hyperkeratosis, several macula and papules on neck and armpits for one-month duration (Fig. 1). The other patient was a 49 yr old male with diabetes mellitus disorder with severe rash, hyperkratosis, and itching over his thigh, buttock and legs (Fig. 2). The patients were referred to Iran-Zamin Medical Diagnostic Laboratory, Ahvaz, Southwest Iran in 2015 for fungal examination. Scraping was performed from lesions of the skin and pathologic slide was prepared with 20% KOH. In microscopic examination presented huge infestation of *S. scabiei* in all forms of parasite included adult female, nymph stage and eggs (Figs. 3 and 4). The first patient’s spouse was also infested by *Sarcoptes* in mild clinical signs. The disease was diagnosed as Norwegian scabies and the patients were successfully treated with topical ointment of 5% permethrin for two consecutive weeks.

## Discussion

Norwegian scabies is worldwide and severs contagious dermatitis in immune-compromised patients (4). Overall, 30 patients with crusted scabies were reported in Senegal (5). The age of patients was from 5 to 70 year. Two most common factors responsible for Norwegian scabies disease included 1- using immunosuppressive drugs in autoimmune diseases and 2- malnutrition in children. The other factors were mental disability-Down syndrome, physical debilitation cases, HIV infection and longtime usage of topical corticosteroid ointment. Two of 5 immunosuppressive patients died after diagnosis and the other patients were treated by benzylbanzoate and ivermectin (5). A women with diabetes mellitus infected with *S. scabies* was showed with severity of clinical signs contained severe rash over thorax and all for extremities. Biopsy of her skin revealed high infection with adults, eggs, and larva of *S. scabiei* (6). Mehta et al in 2009 presented Norwegian scabies in a 70 yr old woman with using oral corticosteroid history for many years. The clinical sign of patient was severe pruritus and itching during the nights and all of her family were infected. Cutaneous examination and biopsy revealed numerous *Sarcoptes*, eggs, and larvae in skin (7). Currie et al, treated 20 crusted scabies patients with ivermectin, combined with topical scabicide and keratolytic therapy (8). The Norwegian scabies was also reported from a 42 year old man with HIV infection. The count of CD4 was 87 cells/ml and presented widespread hyperkeratotic lesions in his skin (9).

The clinical features of the disease are induced by infiltration of inflammation cells included eosinophils, lymphocytes and histocytes, hyperproliferation of epidermis, increasing permeability of skin vessels and secretion of several cytokines by T and B lymphocytes. Th1 responses are predominant in ordinary scabies whereas Th2 responses are active in Norwegian scabies (10). In immune disorder disease, immune responses are suppressed and in this condition, huge proliferation of the *Sarcoptes* mite can occur and more than millions mite were appeared in skin lesion (11).

One of the important risk factors to acquire Norwegian scabies infestation is organ transplantation. Organ recipient patients should be taken immunosuppressive drugs such as glucocorticoids for prevention organ transplanted rejection. Current study reported an acute Norwegian scabies infestation in two patients. The first patient was a 55 yr old man who had renal transplantation one year ago and had taken 1000 mg prednisolone for one year. His wife also was infested by the mite but with no Norwegian manifestation clinical sign, because she had normal immunity responses. The second patient had 49 yr old with diabetes mellitus and autoimmune disorder. Glucocorticoides drugs can reduce immune responses, especially in cellular immunity reactions.

## Conclusion

This study is the first report of Norwegian scabies in Khouzestan province Southwest of Iran. Transplant recipient patients who live in contaminated areas with *Sarcoptes* infestation should be considered for rapid diagnosis of *S. scabiei* infestation. Delay in diagnosis may result in bacterial infection and septicemia outcome.

## Ethical considerations

Ethical issues (Including plagiarism, informed consent, misconduct, data fabrication and/or falsification, double publication and/or submission, redundancy, etc.) have been completely observed by the authors.
